# Light-activated azobenzene peptide inhibitor of the PD-1/PD-L1 interaction†

**DOI:** 10.1039/d4cc01249f

**Published:** 2024-07-09

**Authors:** Deanne Hayward, Zoë R. Goddard, Marco M. D. Cominetti, Mark Searcey, Andrew M. Beekman

**Affiliations:** a School of Pharmacy, University of East Anglia, Norwich Research Park, Norwich Norfolk NR47TJ UK A.Beekman@uea.ac.uk

## Abstract

Inhibiting the PD-1/PD-L1 protein–protein interaction is a key immunotherapy for cancer. Antibodies dominate the clinical space but are costly, with limited applicability and immune side effects. We developed a photo-controlled azobenzene peptide that selectively inhibits the PD-1/PD-L1 interaction when in the *cis* isomer only. Activity is demonstrated in *in vitro* and cellular assays.

## Abstract 2

A photoswitch peptide that binds to PD-1/PD-L1 only in the *cis* conformation, allowing for external control of the immune checkpoint interaction.

## Graphical abstract

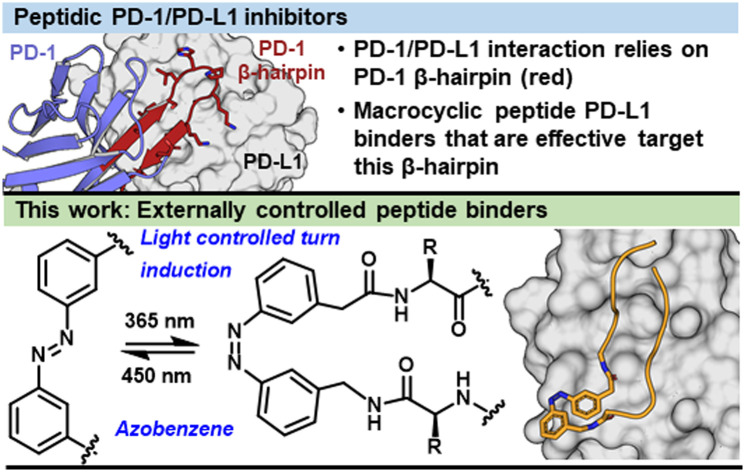

The proliferation of cancer cells relies on evading immune system detection by commandeering immune checkpoint pathways such as the interaction between programmed cell death 1 and programmed cell death ligand 1 (PD-1/PD-L1). Under physiological conditions, the PD-1/PD-L1 pathway negatively regulates T cells to prevent autoimmunity.^1^ By expressing PD-L1 on their cell surface, cancer cells are able to block T cell activation leading to tumour evasion of the immune system.^2^ PD-1/PD-L1 is currently successfully targeted using monoclonal antibodies (mAbs), recovering cancer immunity.^3^ Unfortunately there are several disadvantages found when using mAbs such as poor oral bioavailability, high cost, and immune-related adverse events.^4^ In order to overcome these short falls research has turned to small molecule and peptide inhibitors. Bristol Myers Squibb developed biphenyl small molecule structures that inhibit PD-1/PD-L1 with IC_50_'s in the nanomolar range.^5,6^ Linear peptides that contain a turn structure and cyclic peptides have also been found as effective inhibitors (Fig. 1).^7–13^ However, successful inhibitor candidates have yet to progress through clinical trials to the market.

**Fig. 1:**
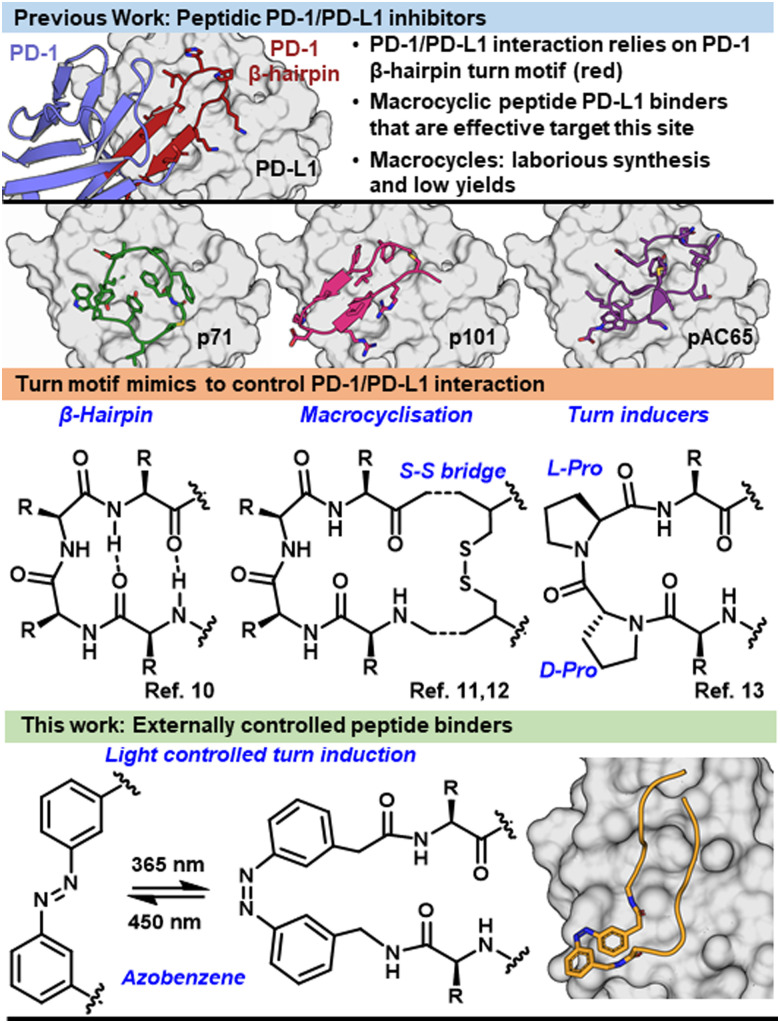
Previously reported peptide binders mimicked a turn motif interaction between PD-1 and PD-L1. Methods to induce turns in peptides include macrocyclization and turn inducing modalities. This work exploited the light controlled turn mimicking ability of azobenzenes.

The binding interface between PD-1/PD-L1 is defined by several β-strands and β-hairpin turn motifs (Fig. 1).^1^ Peptides which mimic this key binding feature of PD-1 have proven advantageous in inhibiting PD-1/PD-L1 (Fig. 1).^7,10,14^ Peptides derived from secondary structures of protein interfaces can be a promising strategy to design protein interaction inhibitors.^15^ However, peptides tend to lose their secondary structure when they are no longer stabilized by the protein domain and therefore exhibit greater conformational freedom. This structural flexibility can make the peptide prone to proteolytic degradation and results in low target affinity due to entropic penalties when binding.^16^ Structural stability can lead to increased binding affinity through displaying hot-spot residues in their bioactive orientation and preventing proteolytic degradation. Methods to increase stability in PD-1/PD-L1 inhibitor design include macrocyclization and turn-inducing amino acids. These approaches modify the backbone of peptides to incorporate a turn structure or force molecular pre-arrangement (Fig. 1).^17–19^

Here, we suggest a strategy to incorporate a small molecule into the backbone of the peptide to align the peptide residues for optimal binding, such as a controlled molecule known as a photo-switch.^20–22^ A photo-switch, such as azobenzene (Fig. 1), allows for reversible switching between two isomeric states, in which, ideally, one of these isomers shows more activity than the other. The incorporation of a photo-switch can act as a controllable turn mimic with the possibility to control potency and site-of-action of a protein–protein interaction inhibitor.^23–25^ The prearrangement of a peptide structure by including the photo-switch allows for the remote control over target-binding affinity,^26–28^ independent from environmental conditions,^29^ allowing for an increased therapeutic window and tumour specific delivery, limiting off target side effects.

The most widely used photo-switches are the azobenzenes due to their fast switching, low photobleaching rate, and straightforward synthesis.^30,31^ Upon irradiation at 365 nm the azobenzene undergoes *trans* to *cis* isomerisation, while relaxing back to the more thermodynamically stable *trans* position thermally or upon irradiation at 450 nm.^31,32^ In the dark at equilibrium, the *trans* conformation of azobenzene is the dominant isomer (>99.99%).^31,33^

Kiora Pharmaceuticals have demonstrated clinical relevance for photo-switchable therapeutics, entering clinical trials for a light-sensing small molecule, KIO-301, to restore sight.^34^ However, this approach is yet to be reported for immune checkpoints.

Peptides can be functionalized with azobenzenes *via* side chain or backbone incorporation, allowing for remote control of the structure.^29^ 3-(3-aminomethylphenylazo)-phenylacetic acid (AMPP) is a known turn mimic in the *cis* isomer, and in approximately half of sequences induces a β-hairpin (Fig. 2B).^23,35,36^ Due to the ability to form a rigid turn when isomerised to the *cis* conformation, AMPP was chosen to insert into the backbone of CLP003 (WHFSYNWRWLPP), a known PD-L1 binder and inhibitor of PD-1/PD-L1, identified with phage display.^37^ We hypothesised that upon irradiation the structural change would induce a turn motif, inducing binding to the target PD-L1 (Fig. 1) and inhibition of the immune checkpoint interaction in a photo-controlled manner.

**Fig. 2:**
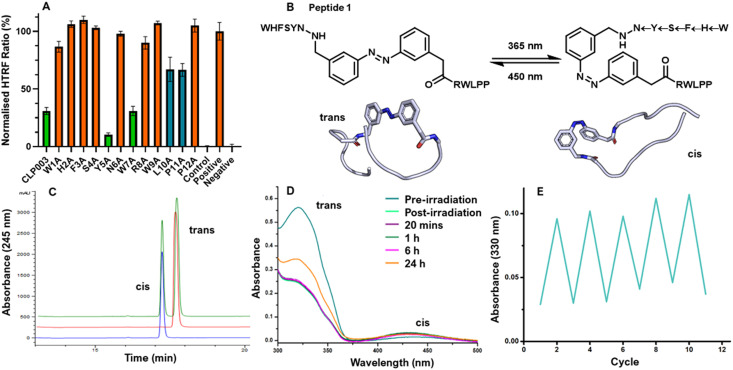
(A) The HTRF assay data for alanine scan of CLP003 showing Y5A and W7A inhibiting PD-1/PD-L1 demonstrating that Tyr5 and Trp7 are not key for binding. All peptides were screened at 1 mM in europium detection buffer, BMS-1 (10 μM) was the control, the positive control contained tagged PD-L1 and tagged PD-1, the negative control contained tagged PD-L1 only. Errors are SEM, performed in triplicate. Representative example of *n* = 3. (B) Example azobenzene modified peptide, peptide 1, predicted to be unstructured in the *trans* isomer and a turn motif in the *cis* isomer. Structures predicted with RFAllAtom and Maestro. The arrows between residues in the *cis* conformation implies the *N* to *C* direction. (C) The analytical HPLC traces (254 nm) showing pre-irradiated (red), irradiated at 365 nm for 20 min (blue) and a co-injection of the two samples (green). (D) UV-Vis spectra of 1. Pre-irradiation demonstrated characteristic maxima for the *trans* isomer at 320 nm. Post irradiation, and after up to 6 hours in the dark characteristic maxima for the *cis* isomer at 450 nm is observed. (E) Absorbance at 330 nm over 10 cycles of photoisomerization of peptide 1 after 20 min irradiation at 365 nm, and then isomerisation for 20 min exposed to white light. After 12 photoswitching cycles the ability to isomerise remained.

Our approach was based on the known PD-1/PD-L1 inhibitor, CLP003 (PD-L1 *K*_D_ = 117 ± 80 nM).^37^ To determine where to insert AMPP, alanine scanning mutagenesis highlighted which amino acids were essential for inhibition using a homogenous time-resolved fluorescence (HTRF) binding assay (Cisbio).^38^ From this Tyr5 and Trp7 were highlighted as being unimportant for inhibition (Fig. 2A). Interestingly, both mutant peptides showed increased inhibition in comparison to the parent peptide with IC_50_ of 0.052 μM [0.028, 0.10] and 0.025 μM [0.013, 0.052] for Y5A and W7A respectively (data in ESI†).

The data collected from the alanine scan of CLP003 was the foundation of the design of azobenzene containing peptides, 1–6 (Fig. 1B and Table 1). AMPP replaced Trp7 in peptide 1, and Tyr5 in AP2. To counteract the size of AMPP, 3 and 4 had Trp7 replaced with AMPP and the Tyr5 removed from the sequence resulting in a peptide of similar size to the parent peptide. Asn was placed on either side of AMPP to account for the displacement of this interaction. We accounted for the changing stereochemistry of Asn due to the removal of amino acids in the sequence and the insertion of AMPP with l-Asn replaced with d-Asn in 5 and 6 to determine which orientation was preferential for inhibition. Fmoc-AMPP was synthesized as previously reported.^39^ All peptides were synthesized using Fmoc solid phase peptide synthesis on TentaGel Rink amide resin with HOBt and HBTU as coupling agents and purified using preparative RP-HPLC. The purity was confirmed using analytical HPLC, and identity confirmed using MALDI-TOF MS.

**Table 1:** The designed azobenzene substituted peptides

Peptide	Sequences
1	Ac-WHFSYN-AMPP-RWLPP-NH_2_
2	Ac-WHFS-AMPP-NWRWLPP-NH_2_
3	Ac-WHFS_N-AMPP-RWLPP-NH_2_
4	Ac-WHFS-AMPP-_NRWLPP-NH_2_
5	Ac-WHFS_n-AMPP-RWLPP-NH_2_
6	Ac-WHFS-AMPP-_nRWLPP-NH_2_

The photo-switching of peptides 1–6 was analysed to assess suitability as a photo-switch. Peptides 1–6 were dissolved 1 mg mL^−1^ in 1 : 1 MeCN/H_2_O and exposed to ambient light. The analytical HPLC of 1 demonstrated the peptide was predominantly in the *trans* isomer, confirmed with UV-Vis spectroscopy (Fig. 2C). After 20 minutes irradiation at 365 nm, the *cis* isomer is predominant. A similar trend was observed for peptides 2–6 (see ESI†).

Determining the stability of peptides 1–6 to remain in the *cis* isomer was essential as this configuration would be the turn required for inhibition of PD-1/PD-L1. The rate of relaxation back to the *trans* isomer was monitored using UV-Vis spectroscopy. Two absorption bands were seen correlating to the two isomers, *trans* (320 nm) and *cis* (450 nm). Upon exposure to ambient light, the *trans* isomer is more dominant than the *cis* (Fig. 2D). After irradiation for 20 minutes at 365 nm, an increase of the *cis* isomer was observed by a decrease in the intensity of the band at 320 nm and an increase in the band at 430 nm. Post-irradiation, peptides 1–6 were kept in the dark and the UV-Vis recorded at time intervals to show that after 24 hours the peptides had still not relaxed back to where the *trans* isomer was the most abundant (Fig. 2D), and after 6 h negligible change in the concentration of the *cis* isomer was observed.

To ensure the peptides were capable of photo-cycling, peptides 1–6 were repeatedly illuminated at 365 nm and white light for 20 minutes under continuous monitoring of UV absorbance band at 330 nm. All peptides yielded a consistent reduction at 330 nm and increase after exposure to white light over 12 cycles, demonstrating photostability as seen with AP1 (Fig. 2E). To determine the ability of each peptide to inhibit the interaction between PD-1/PD-L1 the HTRF assay was used. Each peptide was dissolved in detection buffer with 5% DMSO. A sample was left in the dark and another sample was irradiated at 365 nm for 20 min to obtain the respective *cis* isomer. 1 showed convincing data of the ability to inhibit the interaction with a dose response curve after irradiation determining an IC_50_ of 79 nM [61, 103], while showing no appreciable inhibition in the *trans* isomer at up to 100 μM (Fig. 3A). 1 demonstrated improved interaction inhibition compared to CLP003 (IC_50_ = 4.68 μM [3.55, 6.13], Fig. 3A) and comparable activity to BMS-1 (76 nM).^40–42^ Peptides 2–5 showed no inhibition of PD-1/PD-L1.

**Fig. 3:**
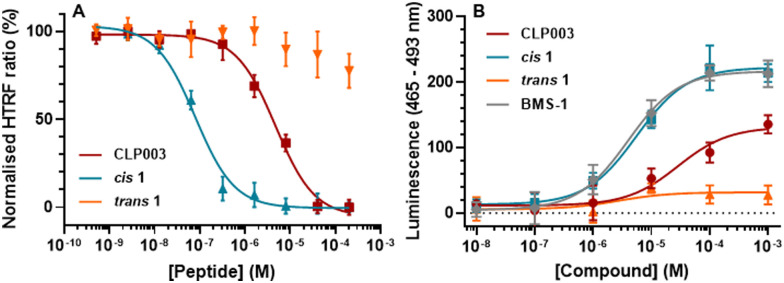
(A) HTRF assay with PD-1/PD-L1 and peptides CLP003, *cis*1 And *trans*1. (B) PD-1/PD-L1 immune checkpoint blockade assay (Invivogen). Increased luminescence implies PD-1/PD-L1 inhibition between modified Jurkat and Raji cells. Errors are SEM, performed in triplicate. Representative examples of *n* = 3.

There was concern that during the cellular assay incubation at 37 °C may result in the azobenzene modified peptide relaxing back to the *trans* isomer. To determine whether 1 was thermally stable to continue with cellular assays, 1 was incubated at 37 °C for 8 h after irradiation at 365 nm for 15 minutes in buffer. The peptide was monitored every hour *via* analytical HPLC and showed no degradation while remaining predominantly the *cis* isomer over 8 h. To explore the activity of 1 in a cellular context, an immune checkpoint blockade assay was performed. The cell-based assay (InvivoGen) consists of Jurkat T cells that are engineered to express luciferase *via* T cell receptor (TCR) signalling and Raji cells engineered to express PD-L1 on the cell surface. If PD-L1 is bound to PD-1, the luciferase expression is inhibited. If the PD-1/PD-L1 interaction is inhibited, TCR signalling causes luciferase expression. As such, luciferase activity directly correlates to the PD-1/PD-L1 interaction, measured by luminescence following luciferin addition. Both *cis* and *trans* isomers of 1 were tested alongside CLP003 and BMS-1 (Fig. 3). At higher concentrations the *cis* isomer of 1 exhibits comparable inhibitory activity of PD-1/PD-L1 to BMS-1, with EC_50_ values of 5.58 μM [3.25, 9.19] and 3.99 μM [2.32, 6.72] respectively. The *cis* isomer of 1 demonstrates a greater inhibitory effect than the parent peptide, CLP003. These data confirm that upon irradiation, the *cis* isomer inhibits PD-1/PD-L1 more effectively than the *trans*.

Peptide immunotherapy treatments offer several benefits to overcome the limitations of mAbs. Photo-switchable peptides that have the potential to be switched “on” and “off” through structural changes, present an opportunity for externally controlled immune checkpoint inhibitors. The combination of peptides and light controlled activity may reduce adverse immune events when compared to antibody therapies which possess immune activating motifs unrelated to the PD-1/PD-L1 pathway. Here, a photo-switchable peptide has shown selective inhibition of PD-1/PD-L1 in the *cis* isomer, demonstrating a nM IC_50_ in a HTRF assay. In a cellular context the *cis* isomer of 1 showed greater inhibition of PD-1/PD-L1 than the *trans* isomer and the parent peptide, CLP003. With a penetrating depth of 60 μM, this UV light activated inhibitor would be beneficiary to treat abnormal cells in the body that a light source could reach such as skin, eyes, mouth, oesophagus, and lungs,^43^ or conditions that are often treated with photodynamic therapy including Bowen's disease, basal cell carcinoma and oesophageal cancer.^44^1 surpassed known peptide binder CLP003 which demonstrates a *K*_D_ of 117 nM.^37^ Our results suggest 1 has comparable activity to macrocyclic peptides of interest BMS-p57 and BMS-p71.^45^ Recently, Liu *et al.* developed a photo-caged prodrug by modifying the PD-1/PD-L1 inhibitor BMS-1 with a photo-removable protecting group, [(diethylamino)coumarin-4-yl]methyl, that can be cleaved upon irradiation at 420 nm. The IC_50_ of the restored inhibitory effect following light irradiation was 1.032 μM.^46^ The approach demonstrated here has improved this proof-of-concept by an order of magnitude and offers great promise in the development of photo-controlled inhibitors of PD-1/PD-L1.

DH: conceptualization, methodology, validation, analysis, data curation, investigation, writing. ZRG: conceptualization, methodology, writing, supervision. MMDC: methodology, supervision. MS: Resources, writing, supervision, project administration. AB: conceptualization, data curation, writing, visualization, supervision, project administration, funding acquisition.

The authors acknowledge support from Big C Cancer Charity (19-13R) and the Royal Society (RGS\R1\201008). Mass spectrometry was supported by BBSRC (BB/T017708/1).

## Data availability

The data supporting this article have been included in the ESI.†

## Conflicts of interest

There are no conflicts to declare.

## Supplementary Material

CC-060-D4CC01249F-s001
