# Supplementary material for: Light-activated azobenzene peptide inhibitor of the PD-1/PD-L1 interaction
Source: Chem Commun (Camb). 2024 Jul 9;60(63):8228–31. doi: 10.1039/d4cc01249f (PMC11293026; doi:10.1039/d4cc01249f)
Supplement: CC-060-D4CC01249F-s001 [file CC-060-D4CC01249F-s001.pdf]

Electronic Supplementary Material (ESI) for ChemComm.
This journal is © The Royal Society of Chemistry 2024

Supporting Information

Light-Activated Azobenzene Peptide Inhibitor of the PD-1/PD-L1 Interaction

Deanne Hayward,[a] Zoë R. Goddard,[a] Marco M.D. Cominetti,[a] Mark Searcey,[a] and Andrew M. Beekman*[a]

School of Pharmacy, University of East Anglia, Norwich Research Park, Norwich, Norfolk, NR47TJ, UK.

E-mail: A.Beekman@uea.ac.uk.

Contents

1. General procedures..........3
Reagents and solvents..........3
Physical characterisation and spectroscopic techniques..........3
Chromatographic techniques..........3
2. Synthesis of Fmoc-AMPP..........4
3. General procedure for peptide synthesis..........6
CLP003 and alanine substituted peptides..........6
1-6..........11
4. Photo-switching of 1-6..........12
1..........12
2..........13
3..........13
4..........14
5..........14
6..........14
5. Thermal stability of 1..........15
6. PD-1/PD-L1 HTRF binding assay..........15
7. PD-1/PD-L1 cell-based assay..........16
8. Structure Modelling..........17
9. References..........17

1. General procedures

Reagents and solvents

All chemicals were reagent grade and were purchased from Sigma Aldrich, Fisher Scientific and Fluorochem. Fmoc-amino acids and coupling reagents were purchased from Novabiochem or Fluorochem. Anhydrous solvents were bought from Sigma Aldrich and assumed to conform to specification.

Physical characterisation and spectroscopic techniques

^{1}H- and ^{13}C-NMR spectra were recorded on a Bruker spectrometer operating at 400 MHz (^{1}H) or 101 MHz (^{13}C) using the specified deuterated solvent. The chemical shifts for both ^{1}H- and ^{13}C- were recorded in ppm and were referenced to the residual solvent peak of either $CHCl_3$ at 7.26 ppm (^{1}H) and 77.0 ppm (^{13}C) or DMSO at 2.50 (^{1}H) and 39.5 ppm (^{13}C). Multiplicities in the NMR spectra are described as s = singlet, d = doublet, t = triplet, q = quartet, m = multiplet, br = broad, and combinations thereof; coupling constants are reported in Hz. Low resolution mass was recorded using an Agilent 1200 Series equipped with a 6460 triple quadrupole. MALDI-MS was performed on a Kratos Analytical Axima-CFR MALDI ToF, using cyano-4-hydroxycinnamic as the matrix. Melting points were recorded using open capillary tubes in a melting point apparatus, melting points are uncorrected.

Chromatographic techniques

Analytical RP-HPLC was performed using an Agilent 1200 HPLC, as described for each peptide. Preparative RP-HPLC was performed using an Agilent 1200 HPLC, fitted with an Agilent eclipse XDB-C18 column (21.2 x 150 mm, 5 µM) and a flow rate 20 mL/min. Spectra were run with a solvent gradient of 0-100% B over 15 min. Solvent A: 95% H_2O, 5% MeCN, 0.05% TFA, solvent B: 95% MeCN, 5% H_2O, 0.05% TFA. Detection wavelengths were 214 nm and 254 nm.

2. Synthesis of Fmoc-AMPP

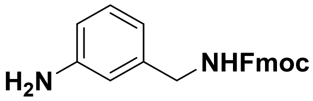

S1

Compound **S1** was prepared following a published protocol.[2] A solution of Fmoc-OSu (3.4 g, 10 mmol) in acetonitrile (25 mL) was added dropwise to a mixture of 3-aminobenzylamine (1.2 g, 10 mmol) and triethylamine (1.4 mL/1.0 g, 10 mmol) in acetonitrile/DMF (10:1, 22 mL). After 1 h of stirring at room temperature, the reaction was precipitated with water to form a white solid. The precipitant was filtered and washed with diethyl ether/trifluoroethanol (1:1, 30 mL) to form a white solid (1.92 g, 5.57 mmol, 56%). The data matched that observed in the literature.[2] Mp: 142-143 °C. ^{1}H NMR (400 MHz, DMSO-d_6) δ 7.89 (d, J = 7.5 Hz, 2H), 7.76 (t, J = 6.2 Hz, 1H), 7.71 (d, J = 7.4 Hz, 2H), 7.42 (t, J = 7.4 Hz, 2H), 7.33 (t, J = 7.5 Hz, 2H), 6.94 (t, J = 7.7 Hz, 1H), 6.46 (m, 1H), 6.42 (d, J = 8.1, 1H), 6.38 (d, J = 7.5 Hz, 1H), 5.01 (s, 2H), 4.30 (d, J = 7.0 Hz, 2H), 4.22 (t, J = 6.9 Hz, 1H), 4.04 (d, J = 6.1 Hz, 2H).^{13}C NMR (101 MHz, DMSO) δ 157.0, 148.6, 144.0, 140.8, 140.3, 128.8, 127.7, 127.1, 125.3, 120.1, 114.6, 112.6, 112.5, 65.5, 46.8, 44.1. ESI [M+H]$^+$ *m/z* calc. for $[C_{22}H_{21}N_2O_2]^+$ 345.2; found 345.3.

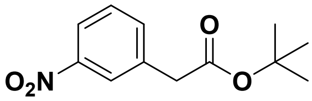

S2

Compound **S2** was prepared following a published protocol.[2] To a solution of 3-nitrophenylacetic acid (2.0 g, 10 mmol) in *tert*-butanol (50 mL) was added Boc_2O (4.8 g, 22 mmol) and DMAP (0.4 g, 3.2 mmol). After 1 hour, the solvent was removed under vacuum leaving a dark brown oil. The crude product was redissolved in DCM and purified using a silica plug with 10% ethyl acetate in hexane to yield a yellow oil (quant.). The data matched that observed in the literature.[2] ^{1}H NMR (400 MHz, $CDCl_3$-*d*) δ 8.13 (s, 1H), 8.09 (t, J = 8.2 Hz, 1H), 7.59 (d, J = 7.6, 1H), 7.47 (t, 7.8 Hz, 1H), 3.62 (s, 2H), 1.43 (s, 9H). ^{13}C NMR (101 MHz, $CDCl_3$) δ 169.7, 148.3, 136.6, 135.7, 129.4, 124.4, 122.1, 81.7, 42.0, 28.0. ESI-QQQ [M+H]$^+$ *m/z* calc. for $[C_{12}H_{16}NO_4]^+$ 237.1; found 238.2.

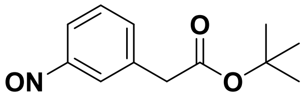

S3

Compound **S3** was prepared following a published protocol.[2] **S2** (2.50 g, 10.5 mmol) was dissolved in 2-methoxyethanol (60 mL) and NH_4Cl (0.85 g, 16 mmol) in 20 mL of H_2O was added. The resulting solution was degassed with argon for 1 h. With vigorous stirring, zinc (2.0 g, 31 mmol) was added over the course of 1 h. The reaction was monitored by TLC (25% ethyl acetate in hexane). After the starting material had disappeared, the reaction was filtered over celite and washed with methoxyethanol (2 x 10 mL). Iron (III) chloride hexahydrate (5.7 g, 21 mmol) in H_2O/EtOH (2:1 vol/vol, 80 mL) was cooled to -10 °C and the filtrate was then added dropwise. After stirring for 1 h, the reaction was poured into ice cold water with vigorous stirring and extracted with ethyl acetate (x 3). The collected organic layers were dried with Na_2SO_4 and concentrated under vacuum to give a green oil that was continued to the next step without purification. The data matched that observed in the literature.[2] Rf 0.23 (25% ethyl acetate in hexane). HRMS $[M+H]^+$ *m/z* calc. for $[C_{12}H_{16}NO_3]^+$ 223.1052; found 223.1096.

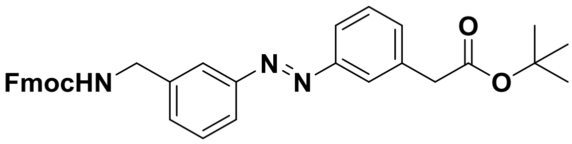

S4

Compound **S4** was prepared following a published protocol.[2] To a solution of **S1** (2.25 g, 10.18 mmol) in acetic acid (50 mL), **S3** (3.5 g, 10.2 mmol) in acetic acid (50 mL) was added dropwise under N_2 and left to stir overnight. The solvent was removed and purified using a silica gel column chromatography (25% ethyl acetate in hexane) to give an orange oil (3.12 g, 5.70 mmol, 56%). The data matched that observed in the literature.[2] Rf 0.25 (30% ethyl acetate in hexane). ^{1}H NMR (400 MHz, $CDCl_3$-*d*) δ 7.87 – 7.81 (m, 4H), 7.76 (d, *J* = 7.6 Hz, 2H), 7.61 (d, *J* = 7.5 Hz, 2H), 7.53 – 7.44 (m, 3H), 7.44 – 7.37 (m, 4H), 7.31 (t, *J* = 7.5 Hz, 2H), 5.24 (bs, 1H), 4.56 – 4.33 (m, 4H), 4.25 (t, *J* = 6.8 Hz, 1H), 3.64 (s, 2H), 1.46 (s, 9H). ^{13}C NMR (101 MHz, $CDCl_3$) δ 170.7, 156.6, 153.0, 152.8, 144.0, 141.5, 140.0, 136.0, 132.1, 130.2, 129.6, 129.3, 127.8, 127.2, 125.2, 123.6, 122.5, 122.0, 121.5, 120.1, 81.3, 66.9, 47.4, 45.0, 42.5, 28.2, 28.0. ESI-QQQ $[M+H]^+$ *m/z* calc. for $[C_{34}H_{34}N_3O_4]^+$ 548.3; found 548.4.

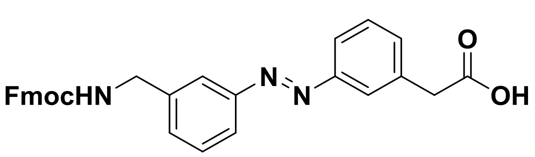

S5

Fmoc-AMPP, **S5**, (3.12 g, 5.70 mmol) was prepared following a published protocol.[2] Compound **S4** was dissolved in DCM (40 mL) and treated with TFA (10 mL) dropwise. The reaction mixture was left to stir overnight under N_2. The mixture was then washed with water until pH was neutral, then washed with brine and dried with Na_2SO_4 before being concentrated under vacuum to give an orange solid (2.7 g, 5.5 mmol, 98%). The data matched that observed in the literature.[2] ^{1}H NMR (400 MHz, DMSO-d_6) δ 12.45 (bs, 1H), 8.00 (t, J = 6.2 Hz, 1H), 7.89 (d, J = 7.4 Hz, 2H), 7.81-7.75 (m, 4H), 7.71 (d, J = 7.5 Hz, 2H), 7.58-7.53 (m, 2H), 7.48-7.37 (m, 4H), 7.31 (t, J = 7.5 Hz, 2H), 4.37 (d, J = 6.6 Hz, 2H), 4.32 (d, J = 6.1 Hz, 2H), 4.25 (t, J = 7 Hz, 1H), 3.74 (s, 2H). ^{13}C NMR (101 MHz, DMSO) δ 172.5, 156.4, 152.0, 151.9, 143.9, 141.4, 140.8, 136.6, 132.7, 130.2, 129.4, 129.3, 127.6, 127.1, 125.2, 123.1, 121.6, 121.4, 120.5, 120.1, 65.4, 46.8, 43.5. ESI-QQQ $[M+H]^+$ *m/z* calc. for $[C_{30}H_{26}N_3O_4]^+$ 492.2; found 492.2.

3. General procedure for peptide synthesis

The twelve amino acid CLP003 peptide (Ac-WHFSYNWRWLPP-NH_2) and the azobenzene substituted peptides (**1-6**) were synthesised on TentaGel S RAM resin (resin loading 0.23 mmol/g) using an automated peptide synthesiser.[1] The AMPP unit was coupled identically as other amino acids. Tentagel S Ram resin (100 mg, 0.023 mmol) was suspended in DMF (2 mL) and allowed to swell for 20 minutes. The DMF was drained from the peptide vessel and Fmoc deprotection was carried out by addition of 20% piperidine in DMF (2 mL), which was shaken for 10 minutes, the repeated. This was removed and the resin was washed with DMF (3 x 2 mL). The resin was then treated with a solution of Fmoc-Pro-OH (4 equiv. compared to resin loading), to which HBTU (3.9 equiv.) and HOBt (4 equiv.) and DIPEA (8 equiv.) in DMF were added. The mixture was then shaken for 45 min. The vessel was drained and the resin washed with DMF (3 x 2 mL). The coupling reaction was then repeated followed by Fmoc deprotection (2 mL 20% piperidine in DMF, 10 min, twice) and finally the resin was washed with DMF. Subsequent amino acids were coupled in an identical fashion. After the final amino acid coupling reaction (tryptophan) and Fmoc deprotection, the resin was treated with acetic anhydride (4 equiv.) and DIPEA (3.9 equiv.) in DMF and reacted for 45 minutes. The resin was washed with DCM (x 3) and MeOH (x 3) to remove any residual DMF.

CLP003 and alanine substituted peptides

The general procedure above was followed. Once synthesised the peptide was cleaved from the resin using 94:2.5:2.5 TFA:H_2O:TIPS (5 mL) and shaken for three hours. The resulting

solution was collected and the resin was washed with TFA (3 x 1 mL), the solutions were combined and concentrated under vacuum. The peptide was precipitated using cold diethyl ether and filtered. The peptide was purified using preparative RP-HPLC and concentrated under vacuum to yield a white solid (3.6 mg, 2.1 µmol, 9%). RP-HPLC: t_R = 11.98 min. MALDI-ToF MS $[M+H]^+$ *m/z* calcd. for $[C_{88}H_{109}N_{22}O_{16}]^+$ 1729.83, found 1729.09.

This was repeated for the twelve alanine scanned peptides. Analytical RP-HPLC was performed using an Agilent 1200 HPLC, fitted with an Agilent ZORBAX Eclipse XDB-C8 column (4.6 x 150 mm, 5 µm) and a flow rate of 1 mL/min. Spectra were run with a solvent gradient of 0-80% B over 20 min. Solvent A: H_2O, 0.05% TFA, solvent B: MeCN, 0.05% TFA. Detection wavelengths were 214 nm and 254 nm.:

Ac-AHFSYNWRWLPP-NH_2 (1.7 mg, 1.1 µmol, 4%). RP-HPLC: t_R = 16.92 min. MALDI-ToF MS $[M+H]^+$ *m/z* calcd. for $[C_{80}H_{104}N_{21}O_{16}]^+$ 1614.80, found 1614.83. HRMS (ESI): m/z calculated for $[M + H]^+$ 1614.7964, found 1614.7991.

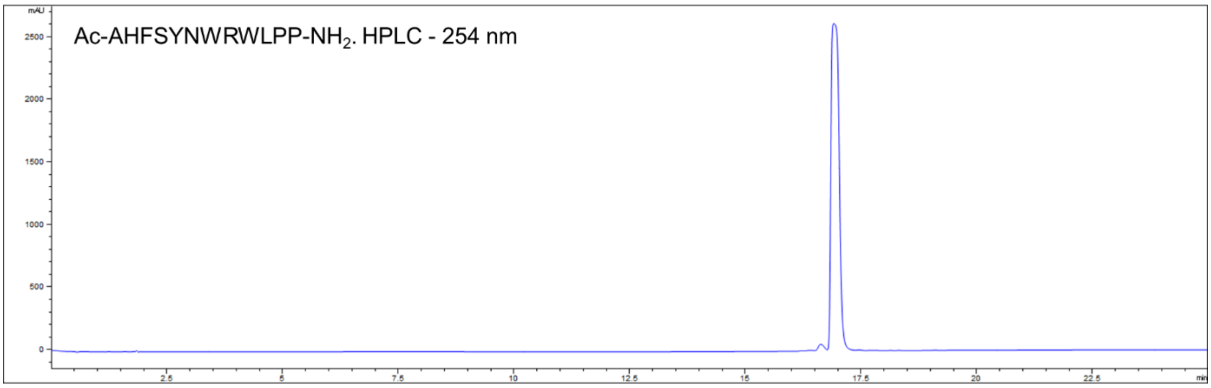

Ac-WAFSYNWRWLPP-NH_2 (2.9 mg, 1.7 µmol, 7%). RP-HPLC: t_R = 17.54 min. MALDI-ToF MS $[M+H]^+$ *m/z* calcd. for $[C_{85}H_{107}N_{20}O_{16}]^+$ 1663.82, found 1663.55. HRMS (ESI): m/z calculated for $[M + H]^+$ 1663.8168, found 1663.8270.

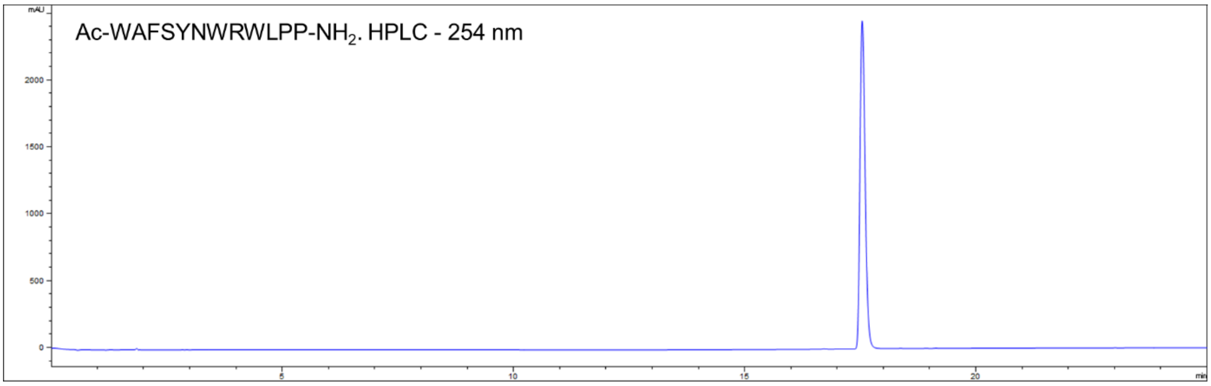

Ac-WHASYNWRWLPP-NH_2 (2.6 mg, 1.6 µmol, 7%). RP-HPLC: t_R = 17.72 min. MALDI-ToF MS $[M+H]^+$ m/z calcd. for $[C_{82}H_{105}N_{22}O_{16}]^+$ 1653.81, found 1653.87. HRMS (ESI): m/z calculated for $[M + H]^+$ 1653.8073, found 1653.8104.

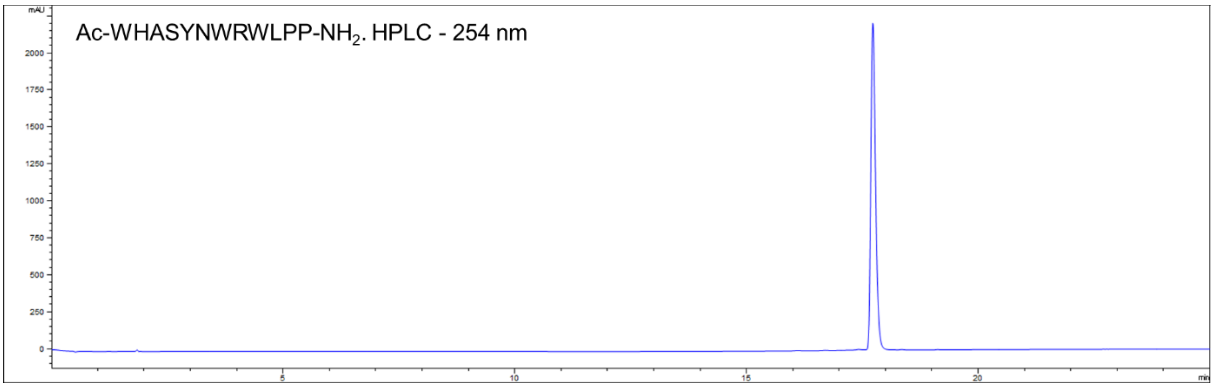

Ac-WHFAYNWRWLPP-NH_2 (2.7 mg, 1.6 µmol, 7%). RP-HPLC: t_R = 16.50 min. MALDI-ToF MS $[M+H]^+$ m/z calcd. for $[C_{88}H_{109}N_{22}O_{15}]^+$ 1713.84, found 1713.64. HRMS (ESI): m/z calculated for $[M + H]^+$ 1713.8437, found 1713.7925.

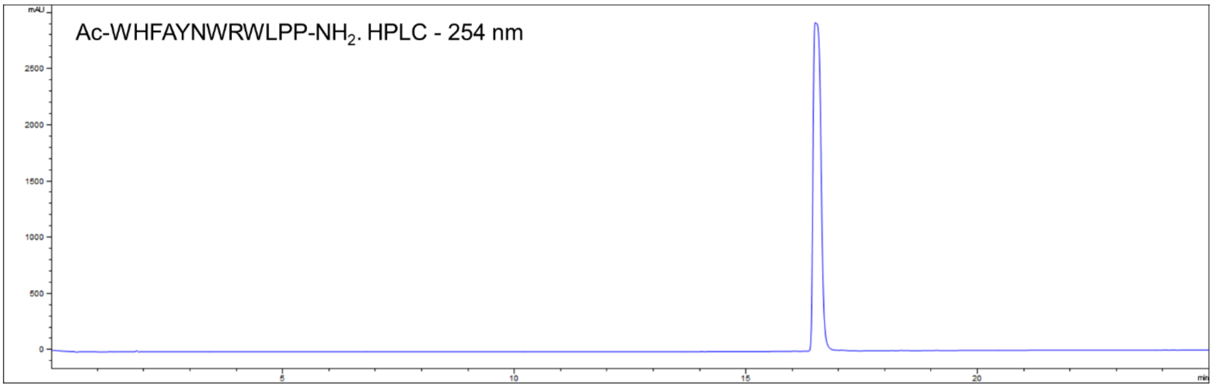

Ac-WHFSANWRWLPP-NH_2 (3.0 mg, 1.3 µmol, 8%). RP-HPLC: t_R = 18.34 min. MALDI-ToF MS $[M+H]^+$ m/z calcd. for $[C_{82}H_{105}N_{22}O_{15}]^+$ 1637.81, found 1637.42. HRMS (ESI): m/z calculated for $[M + H]^+$ 1637.8124, found 1637.8211.

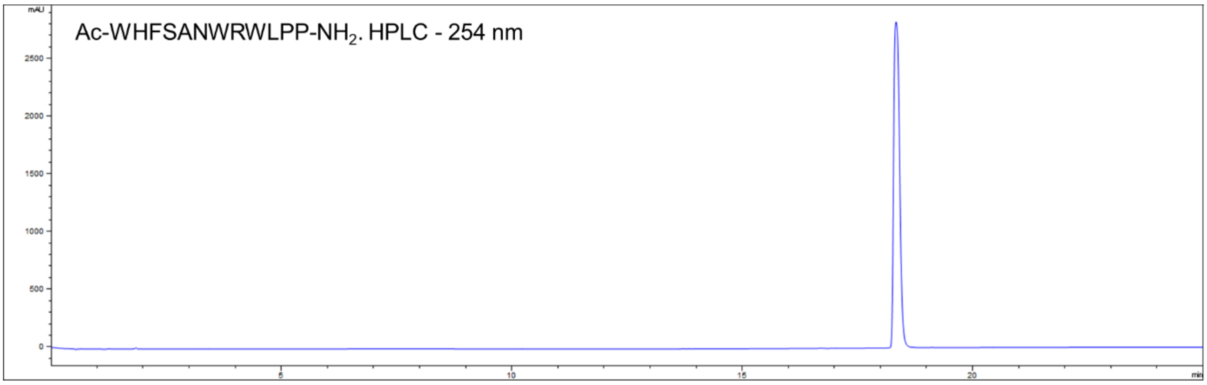

Ac-WHFSYAWRWLPP-NH_2 (4.2 mg, 2.5 µmol, 11%). RP-HPLC: t_R = 18.31 min. MALDI-ToF MS $[M+H]^+$ *m/z* calcd. for $[C_{87}H_{108}N_{21}O_{15}]^+$ 1686.83, found 1686.77. HRMS (ESI): m/z calculated for $[M + H]^+$ 1686.8328, found 1686.8353.

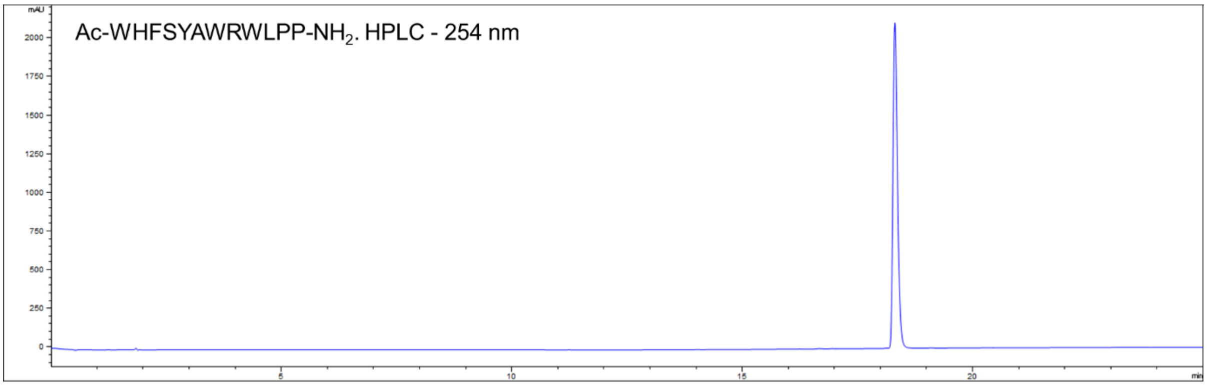

Ac-WHFSYNARWLPP-NH_2 (2.7 mg, 1.7 µmol, 7%). RP-HPLC: t_R = 18.87 min. MALDI-ToF MS $[M-H]^+$ *m/z* calcd. for $[C_{80}H_{104}N_{21}O_{16}]^+$ 1614.80, found 1614.91. HRMS (ESI): m/z calculated for $[M + H]^+$ 1614.7964, found 1614.8168.

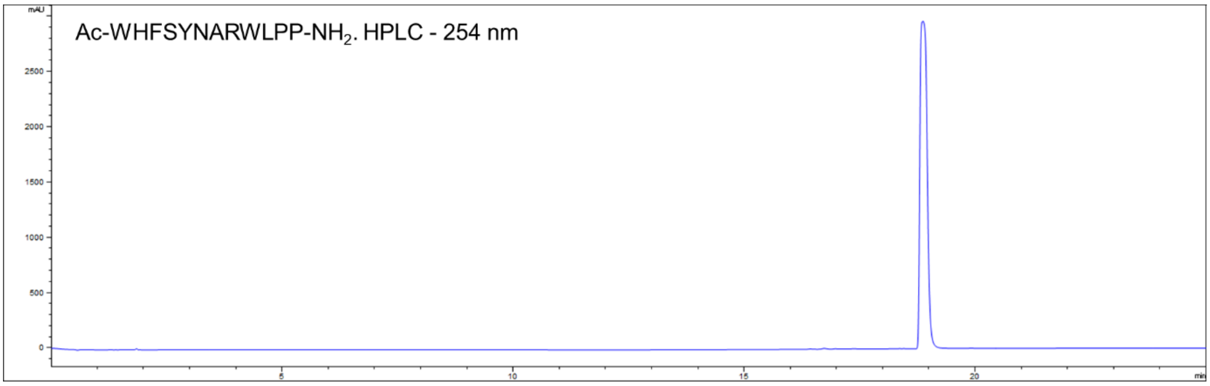

Ac-WHFSYNWAWLPP-NH_2 (1.3 mg, 0.79 µmol, 4%). RP-HPLC: t_R = 19.01 min. MALDI-ToF MS $[M-H]^+$ *m/z* calcd. for $[C_{85}H_{102}N_{19}O_{16}]^+$ 1644.77, found 1644.90. HRMS (ESI): m/z calculated for $[M + H]^+$ 1644.7746, found 1644.7668.

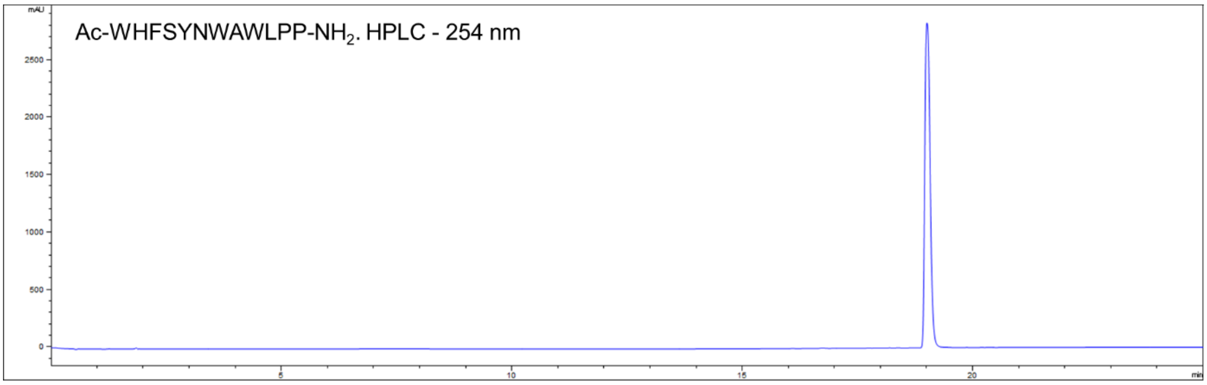

Ac-WHFSYNWRALPP-NH_2 (2.3 mg, 1.4 µmol, 6%). RP-HPLC: t_R = 16.91 min. MALDI-ToF MS $[M-H]^+$ *m/z* calcd. for $[C_{80}H_{104}N_{21}O_{16}]^+$ 1614.80, found 1614.72. HRMS (ESI): m/z calculated for $[M + H]^+$ 1614.7964, found 1614.8011.

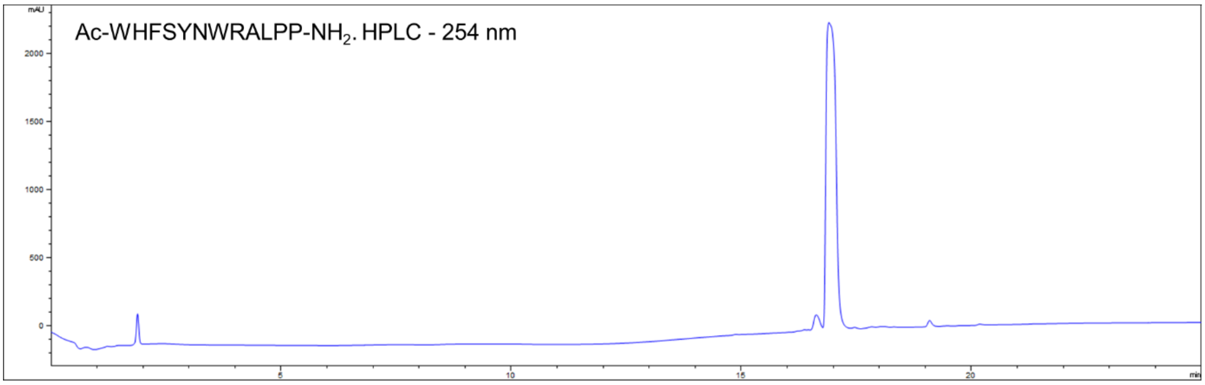

Ac-WHFSYNWRWAPP-NH_2 (1.9 mg, 1.1 µmol, 5%). RP-HPLC: t_R = 17.88 min. MALDI-ToF MS $[M+H]^+$ *m/z* calcd. for $[C_{85}H_{103}N_{22}O_{16}]^+$ 1687.78, found 1687.75. HRMS (ESI): m/z calculated for $[M + H]^+$ 1687.7917, found 1687.7847.

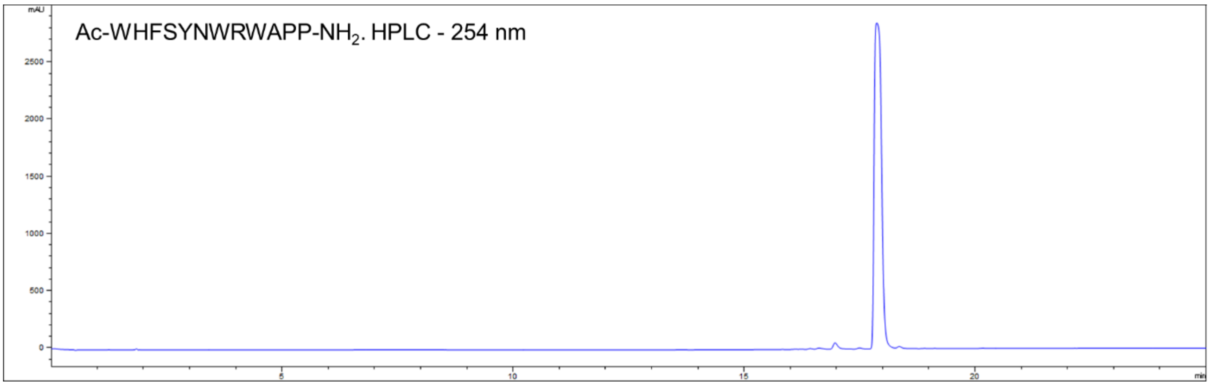

Ac-WHFSYNWRWLAP-NH_2 (2.6 mg, 1.5 µmol, 6%). RP-HPLC: t_R = 18.34 min. MALDI-ToF MS $[M+H]^+$ *m/z* calcd. for $[C_{88}H_{107}N_{22}O_{16}]^+$ 1703.81, found 1703.28. HRMS (ESI): m/z calculated for $[M + H]^+$ 1703.8230, found 1703.8279.

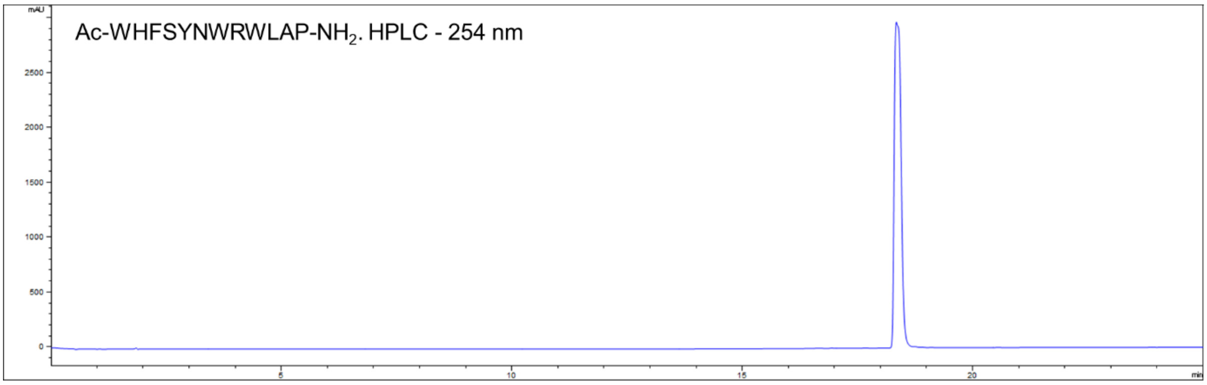

Ac-WHFSYNWRWLPA-NH_2 (3.1 mg, 1.3 µmol, 8%). RP-HPLC: t_R = 18.34 min. MALDI-ToF MS $[M+H]^+$ *m/z* calcd. for $[C_{88}H_{107}N_{22}O_{16}]^+$ 1703.81, found 1703.11. HRMS (ESI): m/z calculated for $[M + H]^+$ 1703.8230, found 1703.8328.

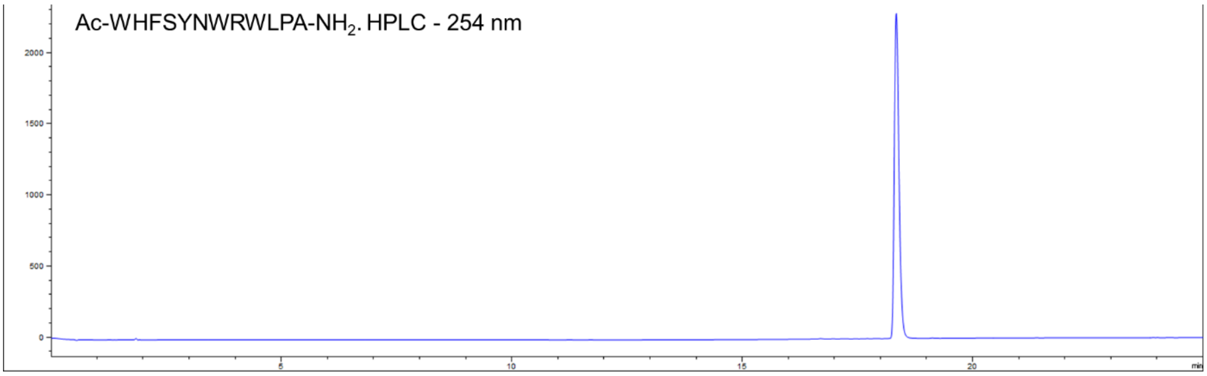

1-6

The general procedure above was followed. Once synthesised the peptide was cleaved from the resin using 94:2.5:2.5 TFA:H_2O:TIPS (5 mL) and shaken for 2 h after which the cleavage cocktail was drained into a round bottom flask. The resin was washed with TFA (3 x 1 mL), the solutions combined and concentrated under vacuum. The peptide was precipitated using cold diethyl ether and filtered. The peptide was purified using RP-preparative HPLC. Analytical RP-HPLC was performed using an Agilent 1200 HPLC, fitted with an Agilent ZORBAX Eclipse XDB-C18 column (4.6 x 150 mm, 5 µm) and a flow rate of 1 mL/min. Spectra were run with a solvent gradient of 0-100% B over 20 min. Solvent A: H_2O, 0.05% TFA, solvent B: MeCN, 0.05% TFA. Detection wavelengths were 214 nm and 254 nm..

1: *trans*-Ac-WHFSYN-AMPP-RWLPP-NH_2 (9.4 mg, 5.2 µmol, 22%). RP-HPLC: t_R = 12.50 min. MALDI-ToF MS $[M+H]^+$ *m/z* calcd. for $[C_{93}H_{116}N_{23}O_{16}]^+$ 1810.90, found 1810.99. HRMS (ESI): m/z calculated for $[M + H]^+$ 1810.8965, found 1810.9011.

2: *trans*-Ac-WHFS-AMPP-NWRWLPP-NH_2 (7.5 mg, 4.5 µmol, 17%). RP-HPLC: t_R = 11.36 min. MALDI-ToF MS $[M+H]^+$ *m/z* calcd. for $[C_{94}H_{113}N_{24}O_{15}]^+$ 1817.87, found 1817.62. HRMS (ESI): m/z calculated for $[M + H]^+$ 1817.8812, found 1817.8790.

3: *trans*-Ac-WHFSN-AMPP-RWLPP-NH_2 (6.2 mg, 3.3 µmol, 16%). RP-HPLC: t_R = 12.50 min. MALDI-ToF MS $[M+H]^+$ *m/z* calcd. for $[C_{84}H_{107}N_{22}O_{14}]^+$ 1647.83, found 1647.66. HRMS (ESI): m/z calculated for $[M + H]^+$ 1647.8332, found 1647.8382.

4: *trans*-Ac-WHFS-AMPP-NRWLPP-NH_2 (6.9 mg, 4.2 µmol, 18%). RP-HPLC: t_R = 12 min. MALDI-ToF MS $[M+H]^+$ *m/z* calcd. for $[C_{84}H_{107}N_{22}O_{14}]^+$ 1647.83, found 1647.49. HRMS (ESI): m/z calculated for $[M + H]^+$ 1647.8332, found 1647.8491.

5: *trans*-Ac-WHFSn-AMPP-RWLPP-NH_2 (4.3 mg, 2.6 µmol 11%). RP-HPLC: t_R = 11.5 min. MALDI-ToF MS $[M+H]^+$ *m/z* calcd. for $[C_{84}H_{107}N_{22}O_{14}]^+$ 1647.83, found 1647.72. HRMS (ESI): m/z calculated for $[M + H]^+$ 1647.8332, found 1647.8306.

6: *trans*-Ac-WHFS-AMPP-nRWLPP-NH_2 (3.8 mg, 2.3 µmol, 10%). RP-HPLC: t_R = 11.4 min. MALDI-ToF MS $[M+H]^+$ *m/z* calcd. for $[C_{84}H_{107}N_{22}O_{14}]^+$ 1647.83, found 1647.90. HRMS (ESI): m/z calculated for $[M + H]^+$ 1647.8332, found 1647.8377.

4. Photo-switching of 1-6

1-6 were dissolved in MeCN/H_2O (1:1 vol/vol) at 1 mg/mL and the analytical HPLC traces were recorded with pre-irradiation showing predominantly *trans* isomer. The samples were exposed to 20 mins irradiation at 365 nm and the analytical HPLC traces were recorded again, showing predominantly the *cis* isomer.

1-6 were dissolved in methanol at 1 µg/mL and the UV-Vis spectra was recorded between 300-500 nm. Samples were exposed to 20 mins irradiation at 365 nm and kept in the dark. UV-Vis spectra was recorded at 20 mins, 1 hour, 3 hours, 6 hours and 24 hours.

1

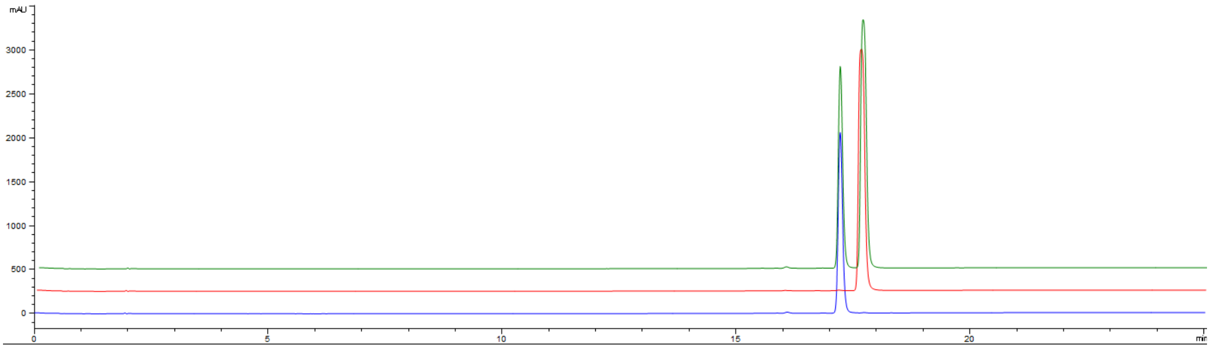

*Figure 1 a) The analytical HPLC traces of **1** reading at 214 nm. Blue: after irradiation at 365 nm for 20 minutes followed by preparative HPLC, showing predominantly the cis isomer (99:1). Red: after being exposed for 20 minutes of ambient light followed by HPLC, showing predominantly the trans isomer (98:2). Green: co-injection. (Agilent eclipse XDB-C18 column (4.6 x 150 mm, 5 µm) and a flow rate of 1 mL/min. Spectra were run with a solvent gradient of 0-75% B over 20 min. Solvent A: H_2O, 0.05% TFA, solvent B: MeOH, 0.05% TFA). B) UV/Vis spectra for **1** after being irradiated for 20 minutes and being kept in the dark while taking time interval readings showing that after 24 hours **1** remains predominantly in the cis isomer.*

2

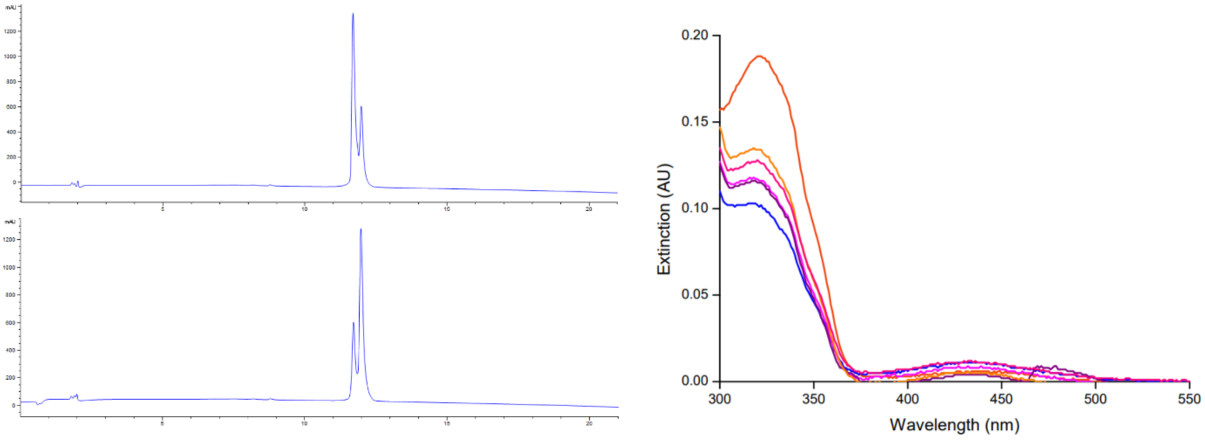

*Figure 2 a) The analytical HPLC traces of **2** reading at 214 nm top: after irradiation at 365 nm for 20 minutes showing predominantly the cis isomer (71:29) bottom: after being exposed for 20 minutes of ambient light showing predominantly the trans isomer (70:30) (Agilent eclipse XDB-C18 column (4.6 x 150 mm, 5 µm) and a flow rate of 1 mL/min. Spectra were run with a solvent gradient of 0-100% B over 20 min. Solvent A: H_2O, 0.05% TFA, solvent B: MeCN, 0.05% TFA). B) UV/Vis spectra for **2** after being irradiated for 20 minutes and being kept in the dark while taking time interval readings showing that after 24 hours **2** remains predominantly in the cis isomer.*

3

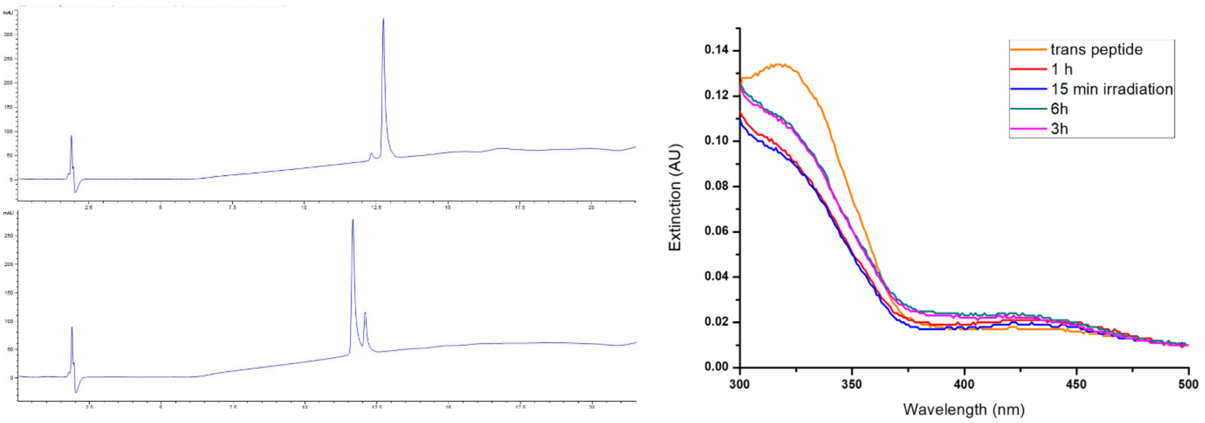

*Figure 3 a) The analytical HPLC traces of **3** reading at 214 nm top: after irradiation at 365 nm for 20 minutes showing predominantly the cis isomer (94:6) bottom: after being exposed for 20 minutes of ambient light showing predominantly the trans isomer (91:9) (Agilent eclipse XDB-C18 column (4.6 x 150 mm, 5 µm) and a flow rate of 1 mL/min. Spectra were run with a solvent gradient of 0-100% B over 20 min. Solvent A: H_2O, 0.05% TFA, solvent B: MeCN, 0.05% TFA). B) UV/Vis spectra for **3** after being irradiated for 20 minutes and being kept in the dark while taking time interval readings showing that after 24 hours **3** remains predominantly in the cis isomer.*

4

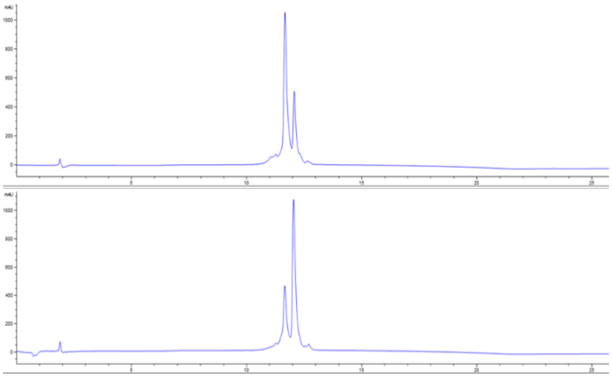

*Figure 4 a) The analytical HPLC traces of **4** reading at 214 nm top: after irradiation at 365 nm for 20 minutes showing predominantly the cis isomer (80:20) bottom: after being exposed for 20 minutes of ambient light showing predominantly the trans isomer (82:18) (Agilent eclipse XDB-C18 column (4.6 x 150 mm, 5 µm) and a flow rate of 1 mL/min. Spectra were run with a solvent gradient of 0-100% B over 20 min. Solvent A: H_2O, 0.05% TFA, solvent B: MeCN, 0.05% TFA). B) UV/Vis spectra for **4** after being irradiated for 20 minutes and being kept in the dark while taking time interval readings showing that after 24 hours **4** remains predominantly in the cis isomer.*

5

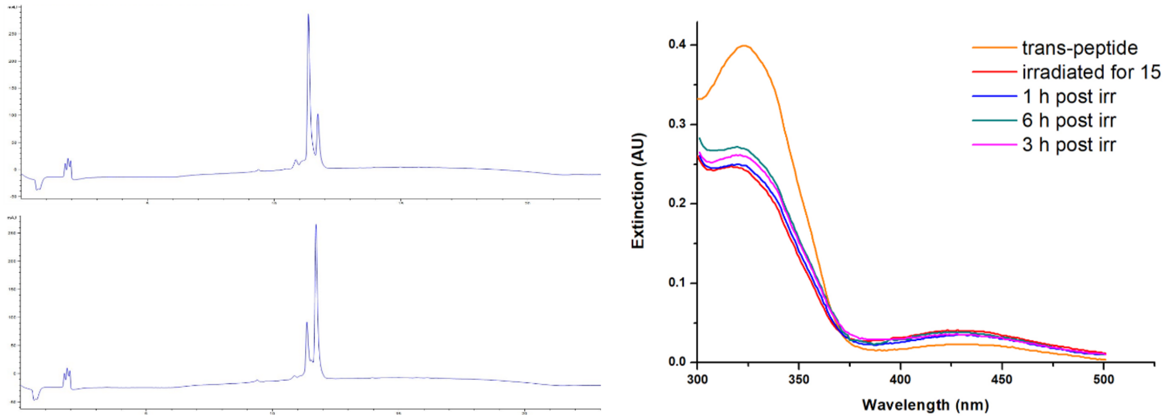

*Figure 5 a) The analytical HPLC traces of **5** reading at 214 nm top: after irradiation at 365 nm for 20 minutes showing predominantly the cis isomer (85:15) bottom: after being exposed for 20 minutes of ambient light showing predominantly the trans isomer (87:13) (Agilent eclipse XDB-C18 column (4.6 x 150 mm, 5 µm) and a flow rate of 1 mL/min. Spectra were run with a solvent gradient of 0-100% B over 20 min. Solvent A: H_2O, 0.05% TFA, solvent B: MeCN, 0.05% TFA). B) UV/Vis spectra for **5** after being irradiated for 20 minutes and being kept in the dark while taking time interval readings showing that after 24 hours **5** remains predominantly in the cis isomer.*

6

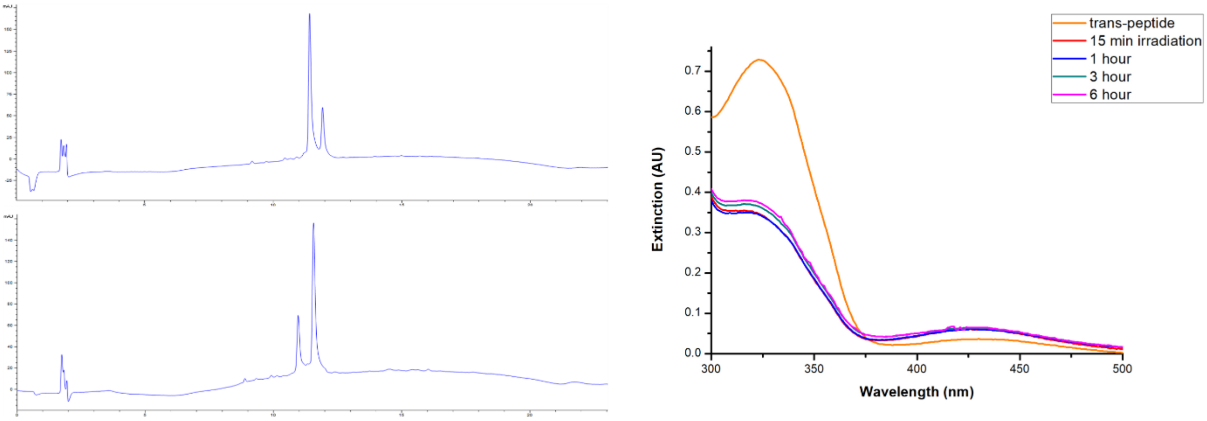

*Figure 6 a) The analytical HPLC traces of **6** reading at 214 nm top: after irradiation at 365 nm for 20 minutes showing predominantly the cis isomer (74:26) bottom: after being exposed for 20 minutes of ambient light showing predominantly the trans isomer (78:28) (Agilent eclipse XDB-C18 column (4.6 x 150 mm, 5 µm) and a flow rate of 1 mL/min. Spectra were run with a solvent gradient of 0-100% B over 20 min. Solvent A: H_2O, 0.05% TFA, solvent B: MeCN, 0.05% TFA). B) UV/Vis spectra for **6** after being irradiated for 20 minutes and being kept in the dark while taking time interval readings showing that after 24 hours **6** remains predominantly in the cis isomer.*

5. Thermal stability of 1

The peptide was dissolved in IMDM (ThermoFisher) with 10% FBS, Pen-Strep (100 µg/mL) and Normocin (100 µg/mL) to 1 mg/mL and incubated at 37 °C for 8 hours. The analytical HPLC trace was recorded every hour, with no observable change.

6. PD-1/PD-L1 HTRF binding assay

This assay was purchased from CisBio and was performed following the manufacturer's protocol. Tag1-PD-L1 and Tag2-PD-1 was diluted 40-fold from the stock solution with PPI-Europium detection buffer. Anti-Tag1 Eu Cryptate reagent and Anti-Tag2 XL665 antibody was diluted 50-fold from the stock solution with PPI-Europium detection buffer. HTRF 96-well low volume white plate was used. The samples were made to the desired concentration and 2 µL was pipetted into the well. The 4 µL of Tag-1-PD-L1 solution (25 nM) was added, followed by 4 µL of Tag2-PD-1 solution (250 nM). 1X Anti-Tag1 Eu Cryptate reagent solution (25 nM|) and 1X Anti-Tag2 XL665 antibody solution (250 nM) were pre-mixed prior to dispensing 10 µL into the well, for a total volume of 20 µL. The plate was sealed and incubated for 1 hour at room temperature. Samples were analysed in triplicate. Once incubated, the plate was read using a CLARIOstar (BMG Labtech) microplate reader following manufacturer's protocols for time resolved fluorescence (excitation filter EX-TR, emission filters 620 (10) nm, 665 (10) nm, dichoric mirror LP-TR, integration delay (lag time) 60 µs, integration time 400 µs, number of flashes 200, gain for white plate 2500).

The emission ratio was calculated for each well was calculated with the following equation:

$$Ratio = \frac{665\,nm\,Intensity}{620\,nm\,Intensity} \times 10^4$$

The "specific signal" (ΔR) is obtained by subtracting the background from the signal of each sample ratio

$$\Delta R = Ratio_{Sample} - Ratio_{background}$$

A decreased ΔR suggests an decreased FRET signal, suggesting a decrease in PD-1/PD-L1 interaction. The data was fit to a three parameter logistic regression model with resulting R^2

values of; Figure 3A (main manuscript) CLP003 = 0.9841, cis 1 = 0.9827, trans 1 = 0.4959, Figure 7 (below) CLP003 Y5A = 0.9869, CLP003 W7A = 0.7902.

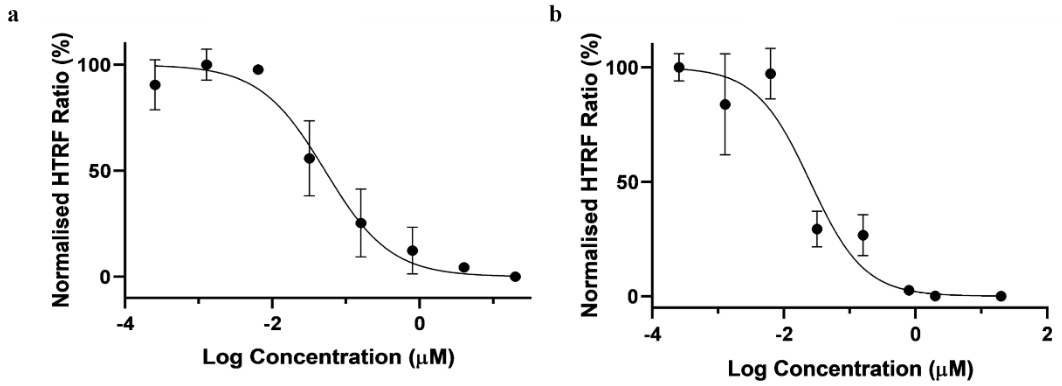

Figure 7 a) The dose dependent curves of CLP003 Y5A with an IC_{50} = 0.052 μM [0.028, 0.010] b) CLP003 W7A with an IC_{50} = 0.025 μM [0.013, 0.052]. Errors are 95% confidence interval.

7. PD-1/PD-L1 cell-based assay

The assay was performed following the manufacturer's protocol. Jurkat-Lucia TCR-hPD-1 cells were cultured in IMDM (ThermoFisher) with 10% FBS, Pen-Strep (100 μg/mL) and Normocin (100 μg/mL). Jurkat cells were passaged every 4 days to a seeding density of 30,000 cells/mL. Every other passage Blasticidin (10 μg/mL), Zeocin (100 μg/mL), Hygromycin (100 μg/mL) and Geneticin (250 μg/mL) were added to maintain selection pressure. Raji-APC-nPD-L1 cells were cultured in IMDM (ThermoFisher) with 10% FBS, Pen-Strep (100 μg/mL) and Normocin (100 μg/mL). Raji cells were passaged every 4 days to a seeding density of 30,000 cells/mL. Every other passage Blasticidin (10 μg/mL) and Geneticin (250 μg/mL) were added to maintain selection pressure. 2 days before analysis Jurkat-Lucia TCR-hPD-1 cells were seeded at 50,000 cells/mL and Raji-APC-nPD-L1 cells were seeded at 40,000 cells/mL in media absent of antibiotics. On the day of analysis Raji-APC-nPD-L1 cell suspension ~100,000 (90 μL) was added to a 96 well plate containing test sample (1.8 μL), followed by Jurkat-Lucia TCR-hPD-1 cell suspension of ~200,000 (90 μL). The plate was incubated at 37 °C in a CO_2 incubator for 6 hours. The co-cultured supernatant was transferred to 96-well clear bottomed white plate. QUANTI-Luc 4 reagent was diluted 20X from the stock solution and 50 μL was added per well. The plate was read using a CLARIOstar (BMG Labtech) microplate reader and used following manufacturer's protocols, reading luminescence at a 4 s start time and 0.1 s reading time. Increased luminescence suggests increased luciferase activity, implying PD-1/PD-L1 inhibition. The data was fit to a three parameter logistic regression model with resulting R^2 values of; Figure 3A (main manuscript) CLP003 = 0.8799, cis 1 = 0.9683, trans 1 = 0.4103, BMS1 = 0.9691

8. Structure Modelling

Predicted structures of peptide 1 unbound and bound to PD-L1 were prepared with RosettaFold All Atom (RFAllAtom),[3] following the protocols provided by the authors (https://github.com/baker-laboratory/RoseTTAFold-All-Atom). Structures were minimized with Maestro from the Schrodinger Suite and images were prepared with Pymol.

9. References

1. H. Liu, Z. Zhao, L. Zhang, Y. Li, A. Jain, A. Barve, W. Jin, Y. Liu, J. Fetse and K. Cheng, *J. Immunotherapy Cancer*, 2019, **7**, 270.

2. A. Aemissegger and D. Hilvert, *Nat Protoc*, 2007, **2**, 161–167.

3. R. Krishna *et al.*, *Science*, 2024, **384**, eadl2528.
